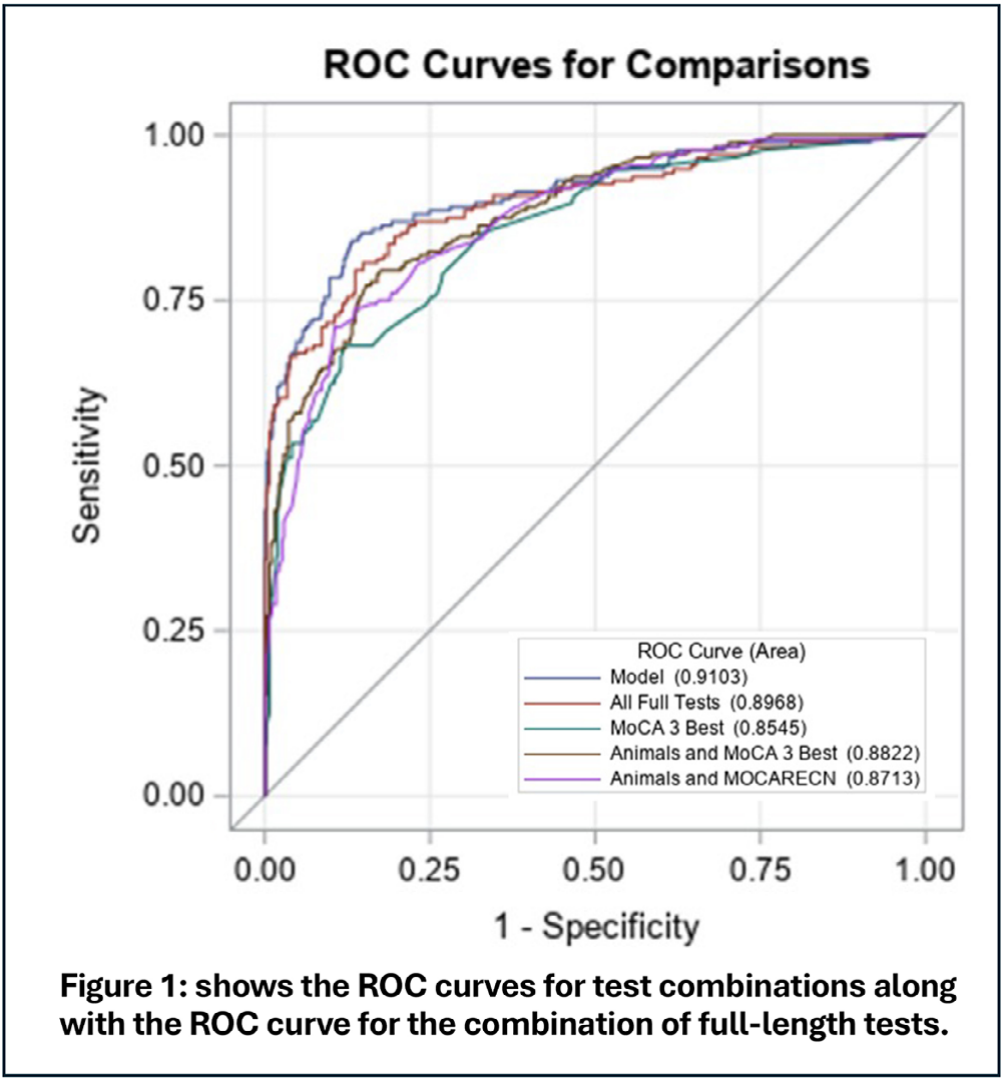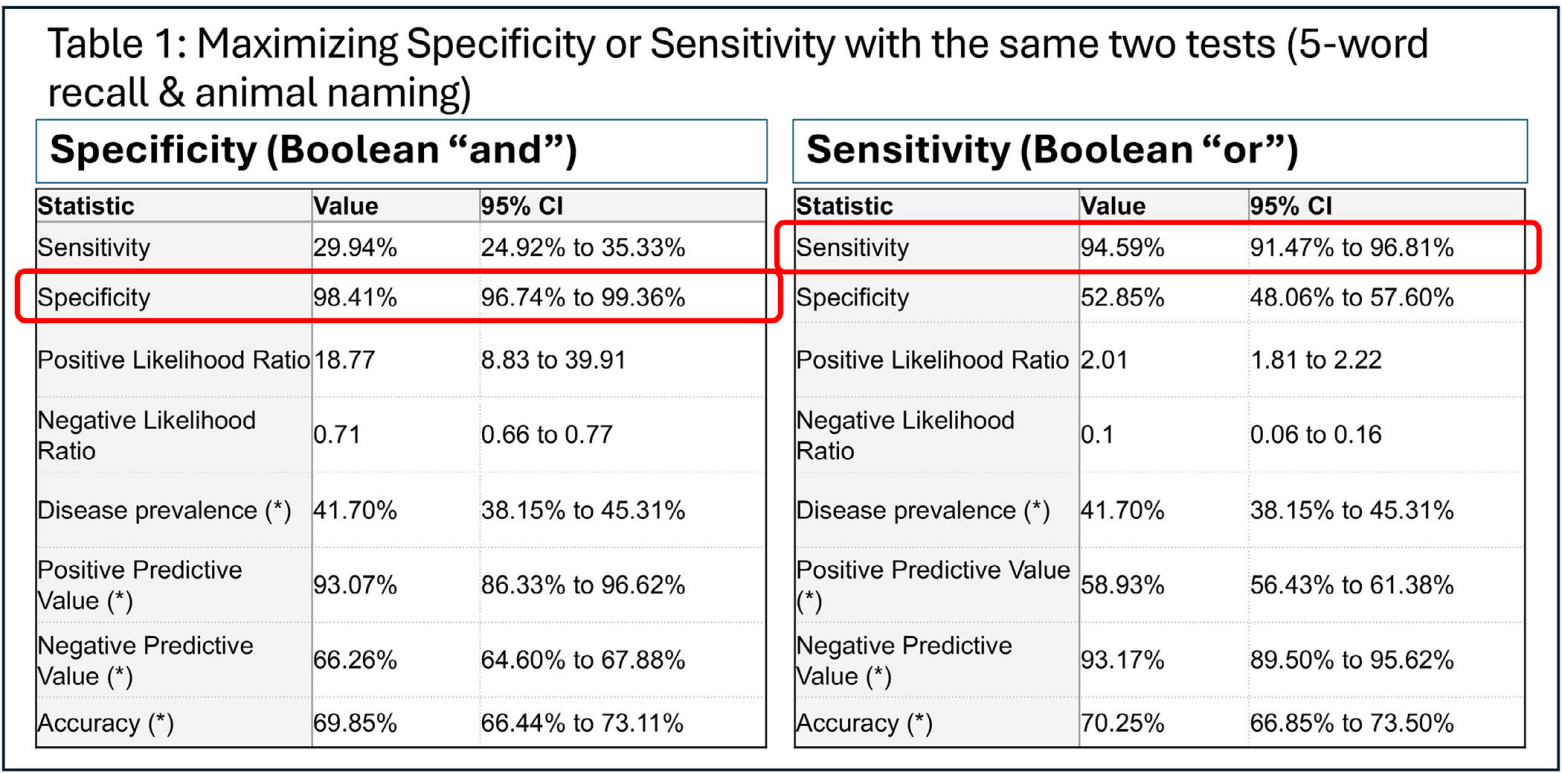# Using 5‐word Delayed Recall and Animal Naming as a highly sensitive and specific, ecologically‐valid, 3‐minute screen for primary care clinics

**DOI:** 10.1002/alz70857_106024

**Published:** 2025-12-25

**Authors:** Elif PINAR Coskun, Shawn T Caudill, Lauren Bojarski, Justin M Barber, Erin L. Abner, Jordan P. Harp, Gregory A Jicha, Christopher J McLouth

**Affiliations:** ^1^ University of Kentucky, SBCoA, Lexington, KY, USA; ^2^ University of kentucky, Department of Neurology, Lexington, KY, USA; ^3^ University of Kentucky, Internal Medicine, Lexington, KY, USA; ^4^ University of Kentucky College of Medicine Department of Neurology, Lexington, KY, USA; ^5^ University of Kentucky College of Medicine, Lexington, KY, USA; ^6^ Sanders Brown Center on Aging, Lexington, KY, USA; ^7^ University of Kentucky College of Medicine Department of Epidemiology, Lexington, KY, USA; ^8^ Department of Epidemiology and Environmental Health, University of Kentucky, Lexington, KY, USA; ^9^ University of Kentucky College of Public Health, Lexington, KY, USA; ^10^ University of Kentucky / Sanders‐Brown Center on Aging, Lexington, KY, USA; ^11^ University of Kentucky Sanders‐Brown Center on Aging, Lexington, KY, USA

## Abstract

**Background:**

Screening for cognitive decline and dementia in primary care practices (PCP) can be burdensome and time consuming, even when using validated and reliable tools such as the MMSE or MoCA. As such, these instruments lack “ecologic validity” (i.e. they are not practical given the time constraints on PCP visits). There is a high‐priority need for less time‐consuming, but still highly sensitive and specific screening tools. The present study explored tests of existing cognitive assessment tools to identify combinations that were optimal for dementia screening in PCP.

**Method:**

Participants from the UK ADRC underwent a full neuropsychological test battery and consensus diagnosis. Logistic regression and receiver operating characteristic (ROC) curves were used to model the probability of conditional on summary test scores and their subcomponents. Youden's J was used to identify cut points that jointly maximized sensitivity and specificity. Boolean logic using “and” and “or” relationships were then used to maximize sensitivity and specificity for this combination of tests.

**Result:**

While using all tests resulted in an AUC of 0.90 (95% CI 0.87 ‐ 0.93)(Fig‐1), this battery takes over an hour to perform. Using only the MoCA 5‐word delayed recall and Animal Naming tests which could be performed in 3 minutes, the AUC was 0.87 (95% CI 0.84 ‐ 0.90). The Youden J cut point indicating cognitive impairment for 5‐word recall was ≤ 2 and for Animal Naming was ≥ 19. Using a Boolean “and” that required both tests to be abnormal resulted in a specificity of 98.41% (95% CI 96.74‐99.36) and a positive predictive value of 93.07% (95% CI 86.33‐96.62). Using a Boolean “or” that required only one of the tests to be abnormal resulted in a sensitivity of 94.59% (95% CI 91.47‐96.81) and a negative predictive value of 93.17% (95% CI 89.50‐95.62)(Table 1).

**Conclusion:**

These data demonstrate that effective dementia screening in the primary clinic can be both practical and highly accurate compared to the gold standard of a full medical and neuropsychological evaluation but can be accomplished in a fraction of the time. Further work validating this screening tool in primary care clinic settings is underway currently.